# Impact of Radiation Therapy on Outcomes of Artificial Urinary Sphincter: A Systematic Review and Meta-Analysis

**DOI:** 10.3389/fsurg.2022.825239

**Published:** 2022-02-14

**Authors:** Li Zhang, Yanwen Xu

**Affiliations:** ^1^Department of Pelvic Floor Comprehensive Diagnosis and Treatment Center, Huzhou Traditional Chinese Medicine Hospital Affiliated to Zhejiang Chinese Medical University, Huzhou, China; ^2^Department of Endocrine, Huzhou Traditional Chinese Medicine Hospital Affiliated to Zhejiang Chinese Medical University, Huzhou, China

**Keywords:** radiotherapy, EBRT, artificial urinary sphincter, prostate cancer, incontinence, meta-analysis

## Abstract

**Background:**

To compare incontinence rates and complications in patients receiving artificial urinary sphincter (AUS) with or without radiotherapy (RT).

**Methods:**

PubMed, Embase, ScienceDirect, CENTRAL, and Google Scholar databases were searched for studies comparing outcomes of AUS between patients with and without RT. Search limits were from 1st January 2002 to 15th September 2021.

**Results:**

Eighteen studies were included. Meta-analysis revealed statistically significant reduced odds of the absence of incontinence in the RT group (OR: 0.35 95% CI: 0.21, 0.59 *I*^2^ = 51% *p* < 0.0001) as compared to the no-RT group. We also noted statistically significant increased risk of revision surgery in the RT group (OR: 1.74 95% CI: 1.16, 2.60 *I*^2^ = 73% *p* = 0.07). There was increased risk of infections (OR: 2.51 95% CI: 1.00, 6.29 *I*^2^ = 46% *p* = 0.05) and erosions (OR: 2.00 95% CI: 1.15, 3.45 *I*^2^ = 21% *p* = 0.01) in the RT group, but the difference was significant only for erosions. Meta-analysis revealed a statistically significant increased risk of explantation in patients with RT (OR: 3.00 95% CI: 1.16, 7.75 *I*^2^ = 68% *p* = 0.02) but there was no difference in the risk of urethral atrophy (OR: 1.18 95% CI: 0.47, 2.94 *I*^2^ = 46% *p* = 0.72) and mechanical failure (OR: 0.90 95% CI: 0.25, 3.27 *I*^2^ = 54% *p* = 0.87) between the two groups.

**Conclusions:**

Our meta-analysis of recent studies indicates that RT significantly reduces the odds of achieving complete continence after AUS placement. History of RT does not increase the risk urethral atrophy or mechanical failure in patients with AUS. However, the risk of revision surgery, erosions and explantations is significantly increased in patients with RT with a non-significant but increased tendency of infections.

**Systematic Review Registration:**

https://www.crd.york.ac.uk/prospero/, identifier: NCT02612389.

## Introduction

Prostate cancer is one of the major causes of cancer-related mortality in men. According to estimates each year around 1.6 million men are diagnosed with and 366,000 men die of prostate cancer (1). An important complication associated with the management of prostate pathologies is urinary incontinence (UI) which can severely impact a patient's quality of life (2). In the long-term, ~9.6% of patients develop UI after 2 years, and the incidence rises to 13.4% at 5 years (3).

UI after prostate treatment can be multifactorial and can be influenced by several variables like patient characteristics, prior continence levels, sphincteric competence, pre and postoperative detrusor function, and surgical techniques (4). While some cases of overactive bladder and UI can be managed by medications, severe UI usually requires management with artificial urinary sphincters (AUS) or urethral slings (5). Since the introduction of AUS in the 1970s, the device has become the surgical gold standard for the management of UI (6, 7). AUS consists of an inflatable cuff that controls the flow of urine by mechanically compressing the urethra. In this context, the importance of baseline healthy tissue cannot be underestimated. One important variable which can influence baseline tissue health is radiation therapy (RT). RT is frequently indicated in cases of prostate cancer to control positive margins or to control extra-prostatic malignancy (8). Furthermore, it is well-known that radiation can lead to significant periurethral tissue damage, fibrosis, reduced vascularity, and poor wound healing (9). Therefore, it is important to understand how does RT changes the success rates and complications of AUS. To the best of our knowledge, only one study by Bates et al. (10) has systematically reviewed the impact of RT on outcomes of AUS. However, an important limitation of their review was that most of their studies were published before 2000. There have been significant advances in surgical procedures and RT protocols over time and there it is unclear how does RT impact outcomes of AUS in the contemporary scenario. Therefore, we designed the current study to assess if prior RT leads to significant deterioration of outcomes of AUS by pooling data only from recent studies.

## Materials and Methods

The methodology of our review was based on reporting guidelines of the PRISMA statement (Preferred Reporting Items for Systematic Reviews and Meta-analyses) (11). The protocol of the review was prospectively registered on PROSPERO (CRD42021274844).

### Literature Search

A systematic and comprehensive search was undertaken on the electronic databases of PubMed, Embase, ScienceDirect, and CENTRAL. We also searched gray literature using Google Scholar (for the initial 200 results of each search query). To minimize single reviewer bias, two authors separately explored the databases. The search limits were set from 1st January 2002 to 15th September 2021. Search terms included were: “radiotherapy,” “radiation,” “EBRT,” “AMS800,” and “Artificial urinary sphincter.” Further details of the search strategy which was common for all databases are presented in Supplementary Table 1. After the initial search, the results were deduplicated and the remaining articles were assessed by their titles and abstracts. We identified studies relevant to the review and extracted their full texts. The two reviewers independently evaluated these studies for final inclusion in the review. Any discrepancies in study selection were resolved by consensus. In the end, manual scoping of the reference list of included studies was carried out for any missed references.

### Eligibility Criteria

Inclusion criteria were defined as follows: (1) All cohort studies, case-control studies, cross-sectional studies, controlled clinical trials, randomized controlled trials comparing outcomes of AUS in patients with or without RT. (2) Studies were to report at least one of the following outcomes: rates of residual incontinence, infections, erosions, explantations, urethral atrophy, or revision surgery. (3) Studies were to report the absolute number of patients with the outcomes. (4) To avoid a small study effect on the outcomes, we included studies with a sample size of >25 patients. No restriction was placed on the etiology or urinary incontinence and all studies comparing outcomes of AUS with and without RT were included.

Exclusion criteria were: (1) Studies on patients undergoing only revision surgeries. (2) Studies including a specific cohort of only compromised patients. (3) Studies not reporting relevant outcomes. (4) Non-English language studies, editorials, review articles. (5) Studies reporting duplicate data. If there were two studies with overlapping data, the study reporting the maximum outcomes was included.

### Data Extraction and Quality Assessment

Two authors independently extracted the following data: author details, publication year, study type, study location, sample size, inclusion/exclusion criteria, age of the patients, percentage of diabetics, the severity of pre-operative incontinence (pads/per day), timing of RT, study outcomes and follow-up. Since residual urinary incontinence was variably measured amongst the included studies, we chose to compare the number of patients with no residual incontinence post AUS placement. Definitions of infection, erosion, explantation, urethral atrophy, and revision surgery were as per the included studies. Since erosions and explantations represent revision surgeries, for studies not reporting data of “revision surgery” *per se*, we included data of erosions/explantations in the meta-analysis for revision surgery.

The methodological quality of studies was assessed using the Newcastle-Ottawa scale (NOS) (12). It was conducted by two authors independent of each other. Any disagreements were solved by a discussion. Studies were assessed for selection of study population, comparability, and outcomes, with each domain being awarded a maximum of four, two, and three points respectively. The maximum score which can be awarded was nine. Studies with nine points were considered to have a low risk of bias, seven to eight points were considered to have a moderate risk of bias and those with scores of six and below were with a high risk of bias.

### Statistical Analysis

The meta-analysis was performed using “Review Manager” (RevMan, version 5.3; Nordic Cochrane Centre [Cochrane Collaboration], Copenhagen, Denmark; 2014). All dichotomous data were pooled using an inverse variance model to calculate odds ratios (OR) with 95% confidence intervals (CI). Meta-analysis was carried out only if at least three studies reported data on the same outcome. All meta-analyses were conducted using the random-effects model. Heterogeneity was assessed using the *I*^2^ statistic. *I*^2^ values of 25–50% represented low, values of 50–75% medium, and more than 75% represented substantial heterogeneity. We assessed publication bias by visual inspection of funnel plots. Funnel plots were created only for analyses including ≥ 10 studies. A sensitivity analysis was carried out to assess the contribution of each study to the pooled estimate by removing one study one at a time and recalculating the pooled effect estimates for the remaining studies. Subgroup analysis was conducted based on the type of RT (Primary RT, post-surgery RT or mixed).

## Results

**Figure 1:**
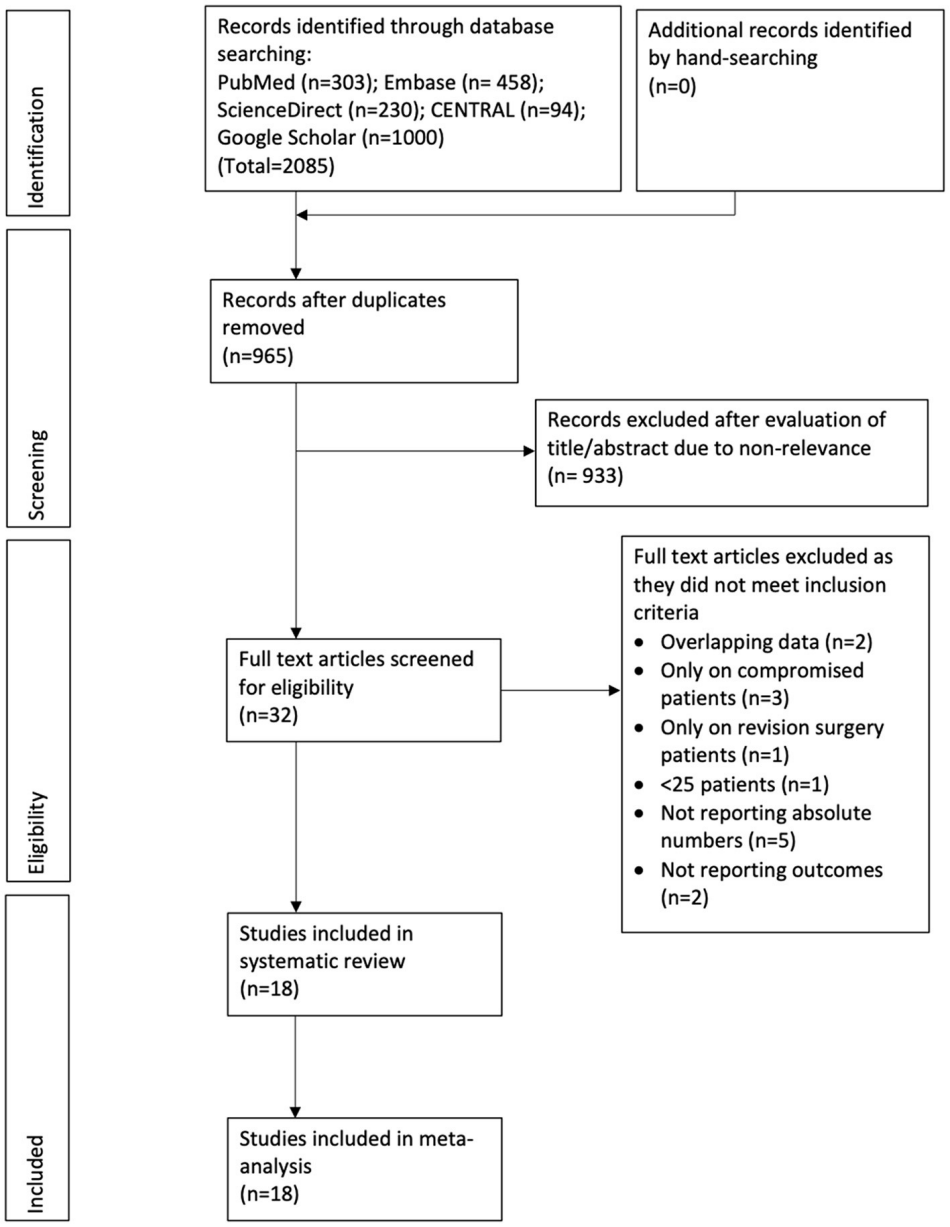
Study flow chart.

The results of the search strategy and the number of records at each stage are presented in Figure 1. A total of 18 studies met the inclusion criteria and were reviewed in our study (13–30). Details of the included studies are presented in Table 1. All included studies were published between 2002 and 2020. The majority of the studies were from the USA. Two (18, 23) were from the UK, two (15, 21) from Germany, one (29) from Australia, and one (28) from France. All were prospective or retrospective cohort studies. The majority of the studies included patients with urinary incontinence post prostate cancer treatment. In three studies (13, 16, 27) all patients with AUS placements were included irrespective of the etiology of incontinence. Amongst studies reporting data, the AMS 800 device was used in most studies. The sample size in the RT group amongst the included studies ranged from 16 to 181 while that of the non-RT group ranged from 28 to 689. The mean age of the patients was above 60 years in all studies. The number of patients with diabetes mellitus ranged from 8.9 to 37.5%. Data on the severity of urinary incontinence was reported by only a small number of studies. When reported, the follow-up was >1 year for all studies.

**Table 1:** Details of included studies.

**Study**	**Location**	**Study type**	**Inclusion criteria**	**Exclusion criteria**	**Device**	**Groups**	**Sample size**	**Age (years)**	**DM (%)**	**Pad per day**	**Follow-up**	**Type of RT**	**Outcomes**
Hird et al. (30)	USA	R	AUS placement following prostate treatment	AUS secondary to neurogenic bladder	NR	RT No-RT	34 39	69.4 67.4	33 39	NR	311.8 days 289.5 days	NR	UI
Sathianathen et al. (29)	Germany	P	AUS placement following prostate treatment	Mild UI, detrusor overactivity apparent during first 300 mL of bladder filling	AMS 800 double cuff	RT No-RT	73 77	69 70	12.3 20.4	7 6.5	24 months	NR	UI, infection, erosion, explantation, MF
Ravier et al. (28)	USA	R	AUS placement following prostatectomy	No prostatectomy or salvage prostatectomy	NR	RT No-RT	46 112	67.2 65.7	8.9 17.8	4.83 4.58	2.6 years 3.8 years	Post-surgery	Infection, erosion, Urethral atrophy, MF
Brant et al. (27)	USA	R	AUS placement following prostatectomy	NR	NR	RT No-RT	152 689	NR	NR	NR	3.7 years	NR	Revision surgery
Sotelo and Westney (26)	UK	P	AUS placement following prostate cancer treatment	NR	NR	RT No-RT	16 28	NR	NR	NR	19 months	Post-surgery and Primary	UI
Rivera et al. (13)	USA	R	All AUS implantations	AUS secondary to neurogenic bladder	AMS 800	RT No-RT	181 308	72 70	19.4 13.7	NR	4.3 years	NR	Revision surgery
Lai et al. (25)	USA	R	AUS placement following prostate cancer treatment	NR	AMS 800,700, 600	RT No-RT	31 63	65.5 63.5	NR	NR	62 months	Post-surgery and Primary	UI, infection, erosion, revision surgery
Raj et al. (24)	USA	R	All AUS placements	NR	NR	RT No-RT	83 93	NR	NR	NR	25-32 months	Post-surgery and Primary	Erosion
Walsh et al. (23)	Germany	R	AUS placement following prostatectomy	NR	AMS 800	RT No-RT	30 64	NR	NR	NR	39.7 months	Post-surgery	Infection
Jahromi et al. (22)	Canada	R	AUS placement following prostatectomy	No prostatectomy	NR	RT No-RT	39 79	NR	NR	NR	NR	Post-surgery	Infection, erosion
Maurer et al. (21)	Australia	R	AUS placement following prostatectomy	No prostatectomy	AMS 800	RT No-RT	29 48	70.9 73	31 37.5	NR	12.2 months	Post-surgery	UI, infection, erosion, revision surgery
Srivastava et al. (20)	France	R	AUS placement following prostate treatment	Neurological or traumatic causes	AMS 800	RT No-RT	61 61	70.1 67	NR	NR	37 months	Post-surgery and Primary	Infection, erosion, explantation, revision surgery
Cohen et al. (19)	USA	P	All AUS placements	NR	NR	RT No-RT	138 248	NR	NR	NR	2.3 years	NR	Explantation
Guillaumier et al. (18)	USA	R	AUS placement following prostate cancer treatment	NR	AMS 800	RT No-RT	22 59	NR	NR	NR	18.8 months	Post-surgery and Primary	Infection, erosion, urethral atrophy, revision surgery, MF
Jhavar et al. (17)	USA	R	AUS placement following prostatectomy	NR	AMS 800	RT No-RT	60 116	70 68.7	NR	5.6 5.2	36.5 months	Post-surgery	Infection, erosion, urethral atrophy, revision surgery, MF
Simhan et al. (16)	USA	R	All AUS placements	NR	AMS 800	RT No-RT	95 542	NR	NR	NR	68 months	NR	Revision surgery
Kretschmer et al. (15)	UK	R	AUS placement following prostatectomy	NR	AMS 800	RT No-RT	22 76	67 69	NR	NR	46 months	Post-surgery	UI, Infection, erosion, urethral atrophy, MF
Gomha and Boone (14)	USA	R	AUS placement following prostatectomy	NR	AMS 800	RT No-RT	28 58	69.7 68.3	NR	NR	32 months	Post-surgery	Infection, erosion, urethral atrophy

*R, retrospective; P, prospective; RT, radiotherapy; UI, urinary incontinence; MF, mechanical failure; NR, not reported*.

### Meta-Analysis

**Figure 2:**
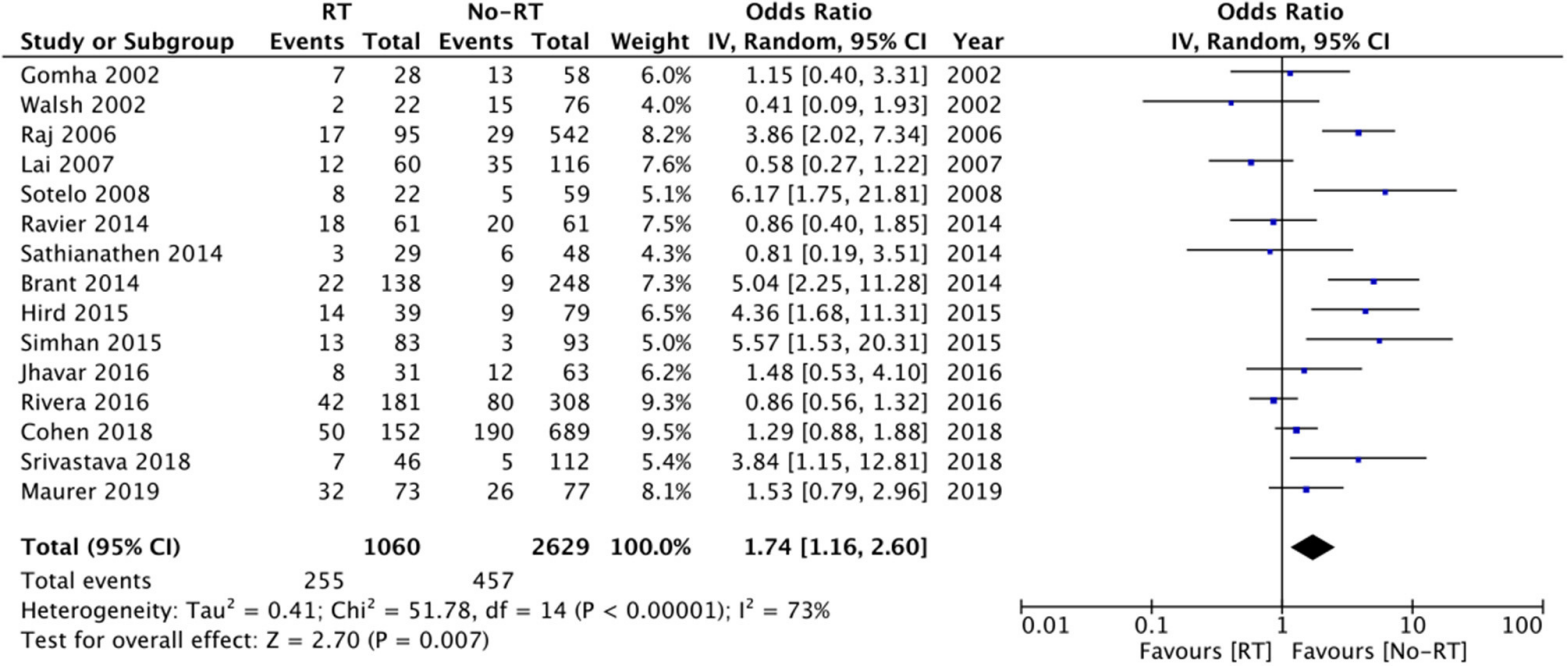
Meta-analysis of no postoperative incontinence between RT and no-RT groups.

We were able to extract data on the absence of urinary incontinence after AUS placement from eight studies (14, 17, 18, 22, 23, 25, 28, 29). Comparing data of 281 patients with a history of RT vs. 489 patients without RT, we noted statistically significant reduced odds of the absence of incontinence in the RT group (OR: 0.35 95% CI: 0.21, 0.59 *I*^2^ = 51% *p* < 0.0001) (Figure 2). Results were stable on sensitivity analysis and there was no change in the significance of the effect size on the exclusion of any study. On subgroup analysis, results were similar for studies including patients with post-surgery RT and mixed population (primary and post-surgery RT) (Table 2).

**Table 2:** Results of subgroup analysis.

**Outcome**	**Groups**	**Number of studies**	**RT (n)**	**No RT (n)**	**Odds ratio**
No postoperative incontinence	Post-surgery RT Mixed	4 2	139 47	298 91	0.34 95% CI:0.12, 0.97 *I*^2^ = 73% *p* = 0.04 0.32 95% CI:0.12, 0.85 *I*^2^ = 26% *p* = 0.02
Revision surgery	Post-surgery RT Mixed	6 4	224 197	489 276	1.29 95% CI:0.57, 2.93 *I*^2^ = 69% *p* = 0.54 2.33 95% CI:0.87, 6.24 *I*^2^ = 71% *p* = 0.09
Infection	Post-surgery RT Mixed	6 3	232 114	477 183	2.55 95% CI:0.57, 11.41 *I*^2^ = 63% *p* = 0.22 2.23 95% CI:0.62, 7.99 *I*^2^ = 20% *p* = 0.22
Erosion	Post-surgery RT Mixed	5 4	202 197	413 276	2.72 95% CI:1.33, 5.55 *I*^2^ = 0% *p* = 0.006 1.55 95% CI:0.38, 6.39 *I^2^* = 56% *p* = 0.54

*CI, confidence interval; RT, radiotherapy; n, number of patients*.

*Mixed group indicates both primary and post-surgery RT*.

**Figure 3:**
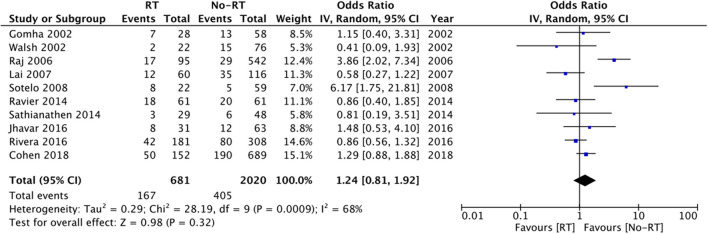
Meta-analysis of revision surgery between RT and no-RT groups.

Data from fifteen studies was pooled for the meta-analysis of revision surgery. Meta-analysis of data from 1,060 patients in the RT group and 2,629 patients in the no-RT group, we noted statistically significant increase in the risk of revision surgery in patients with prior RT (OR: 1.74 95% CI: 1.16, 2.60 *I*^2^ = 73% *p* = 0.07) (Figure 3). There was no evidence of publication bias on visual inspection of the funnel plot (Supplementary Figure 1). On sensitivity analysis, there was no change in the significance of results on the sequential exclusion of any study. On subgroup analysis based on the type of RT, the results indicated a tendency of increased risk of revision surgery in the RT group but the results were statistically non-significant (Table 2).

**Figure 4:**
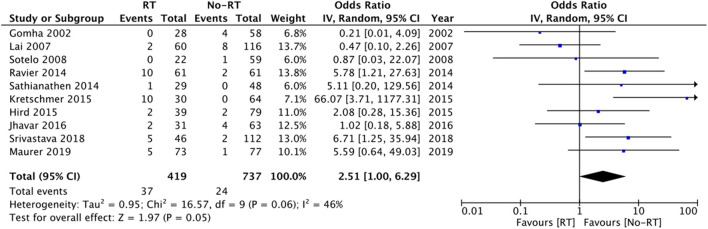
Meta-analysis of infection between RT and no-RT groups.

On comparison of data from 419 patients in the RT group and 737 patients in the no-RT group, we noted a tendency of increased risk of infections in patients with a history of RT but the difference did not achieve statistical significance (OR: 2.51 95% CI: 1.00, 6.29 *I*^2^ = 46% *p* = 0.05) (Figure 4). On the sequential exclusion of data from three studies (14, 17, 25), the results indicated a statistically significant increased risk of infections in patients with RT. There was no evidence of publication bias (Supplementary Figure 2). On subgroup analysis based on the type of RT, the results indicated a tendency of increased risk of infections in the RT group but the results were statistically non-significant (Table 2).

**Figure 5:**
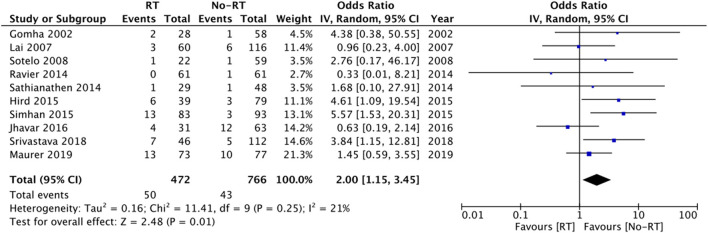
Meta-analysis of erosion between RT and no-RT groups.

Ten studies (14, 16, 17, 20, 21, 25, 26, 28–30) with 472 participants in the RT group and 766 participants in the no-RT group reported data on erosions. The meta-analysis demonstrated that patients with RT have a significantly increased risk of erosions (OR: 2.00 95% CI: 1.15, 3.45 *I*^2^ = 21% *p* = 0.01) (Figure 5). There was no evidence of publication bias (Supplementary Figure 3). On exclusion of the study of Shrivastava et al. (20) the results turned non-significant (OR: 1.79 95% CI: 0.99, 3.23 *I*^2^ = 20% *p* = 0.06). There was no change in the significance of the results on the exclusion of any other study. Subgroup analysis based on type of RT indicated increased risk of erosions in studies including patients with post-surgery RT, however, the difference was non-significant for studies including a mixed patient population (Table 2).

**Figure 6:**
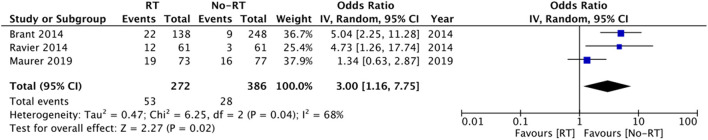
Meta-analysis of explantation between RT and no-RT groups.

**Figure 7:**
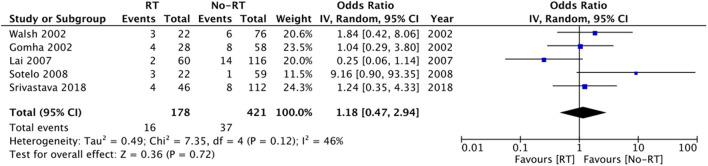
Meta-analysis of urethral atrophy between RT and no-RT groups.

Data on explanation and urethral atrophy was reported by only three (21, 27, 28) and five studies (14, 20, 23, 25, 26) respectively. Meta-analysis revealed a statistically significant increased risk of explantation in patients with RT (OR: 3.00 95% CI: 1.16, 7.75 *I*^2^ = 68% *p* = 0.02) (Figure 6), but there was no difference in the risk of urethral atrophy between the two groups (OR: 1.18 95% CI: 0.47, 2.94 *I*^2^ = 46% *p* = 0.72) (Figure 7). Results of urethral atrophy were stable on sensitivity analysis.

**Figure 8:**
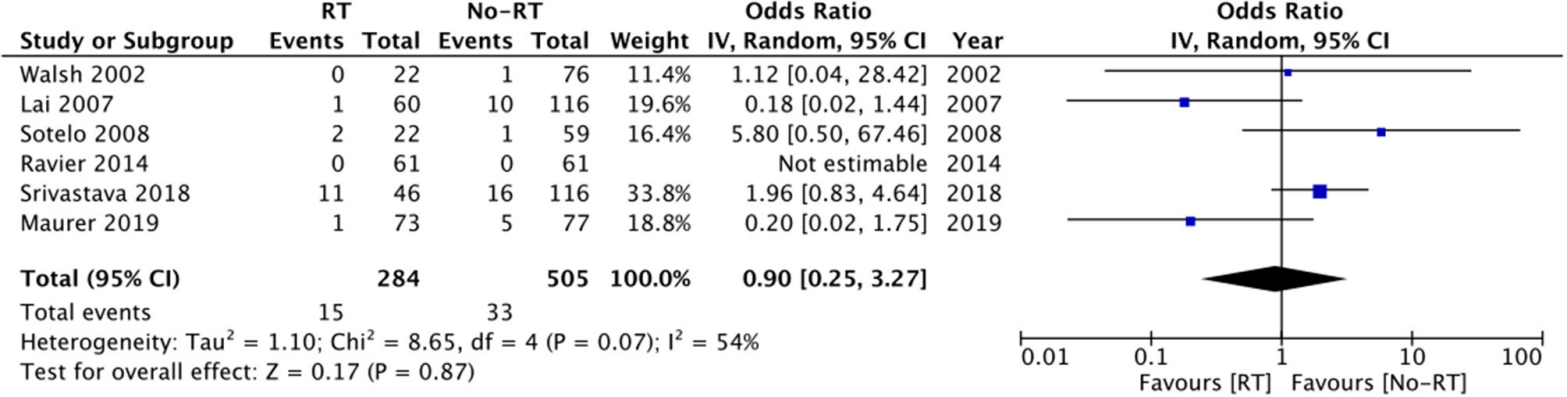
Meta-analysis of mechanical failure between RT and no-RT groups.

Six studies (20, 21, 23, 25, 26, 28) reported data on rates of mechanical failure. Comparing 284 patients in the RT group with 505 in the no-RT group, we found no difference in the risk of mechanical failure between the two groups (OR: 0.90 95% CI: 0.25, 3.27 *I*^2^ = 54% *p* = 0.87) (Figure 8). Results did not change on sensitivity analysis. Subgroup analysis based on type of RT was not conducted for the outcomes of explantation, urethral atrophy, and mechanical failure due to limited number of studies in the analysis.

### Risk of Bias

The risk of bias assessment of included studies based on authors' judgment is presented in Table 3. The NOS scores of the studies ranged from 5–7. There were no high-quality studies.

**Table 3:** Risk of bias analysis based on Newcastle-Ottawa Scale.

**Study**	**Selection**				**Comparability**	**Outcome**			**Total**
	**Representativeness of the exposed cohort**	**Selection of the non-exposed cohort**	**Ascertainment of exposure**	**Demonstration of outcome of interest**	**Basis of the design or analysis**	**Assessment of outcome**	**Follow-up long enough for outcomes**	**Adequate follow up**	
Hird and Radomski (30)	1	1	1	1	0	1	1	1	7
Sathianathen et al. (29)	1	1	1	1	0	1	1	1	7
Ravier et al. (28)	1	1	1	1	0	1	1	0	6
Brant et al. (27)	1	1	1	1	0	1	1	0	6
Sotelo et al. (26)	1	1	1	1	0	1	1	0	6
Rivera et al. (13)	1	1	1	1	0	0	1	0	5
Lai et al. (25)	1	1	1	1	0	1	1	0	6
Raj et al. (24)	1	1	1	1	0	1	1	0	6
Walsh et al. (23)	1	1	1	1	0	1	1	0	6
Jahromi et al. (22)	1	1	1	1	0	1	0	0	5
Maurer et al. (21)	1	1	1	1	0	1	1	0	6
Srivastava et al. (20)	1	1	1	1	0	1	1	0	6
Cohen et al. (19)	1	1	1	1	0	1	1	0	6
Guillaumier et al. (18)	1	1	1	1	0	1	1	0	6
Jhavar et al. (17)	1	1	1	1	0	1	1	1	7
Simhan et al. (16)	1	1	1	1	0	1	1	0	6
Kretschmer et al. (15)	1	1	1	1	0	0	1	1	6
Gomha and Boone (14)	1	1	1	1	0	0	1	0	5

## Discussion

UI is one of the most disturbing adverse events occurring after prostate treatment, be it for benign or malignant conditions. Since its invention in the 1970s, the use of AUS has redefined the management of patients with UI. It provides good continence rates especially in patients after prostatectomy which greatly improves the quality of life and patient satisfaction (6, 7). However, like any device, AUS is also prone to several complications and inadequate success rates. Such complications can be significantly altered by various confounders and one such important variable is RT. Men with prostate cancer frequently require RT for control of extra-prostatic extensions or in case of positive margins (8). Furthermore, as RT is known to have an adverse impact on healthy tissues, it could significantly complicate the outcomes of AUS. Since the introduction of AUS, there have been several studies that have assessed the impact of radiation on outcomes of AUS but with heterogeneous results. In an attempt to provide the best possible evidence and judge the true impact of RT on AUS, the current review was conducted.

The success of AUS is judged by its ability to provide complete continence to the patient. However, according to literature, success rates of AUS vary widely from 59 to 100%, partly due to the difference in the definition of continence and variability of reporting of outcomes amongst studies (31, 32). In the current review, there was a similar heterogeneity amongst the included studies and hence we pooled data on the “absence of UI” at follow-up. This compromise was essential to allow a pooled analysis of data in our review. In our meta-analysis, patients with a history of RT had a statistically significant 65% reduced odds of success with AUS and had persistent UI of some degree after follow-up. This is in concurrence with the results of the previous review of Bates et al. (10) which reported a two-fold increased risk of persistent UI in patients with RT+ prostatectomy vs. prostatectomy alone. Because the majority of the studies in the review of Bates et al. (10) were pre-2001 and only recent studies were included in our review, it suggests that the effect of RT on functional outcomes has persisted despite improvements in RT and treatment protocols over the years. However, these results must be interpreted with caution as standardized scales of measuring UI (31) like pad tests, Patient Global Impression of Improvement score, Urogenital Distress Inventory-6, and Michigan Incontinence Symptom Index score were not routinely used in the studies. There is a need for future studies using such standardized scores to judge the true impact of RT on post-AUS UI outcomes.

The cause of persistent UI after AUS placement in patients receiving RT has been attributed to the radiation-induced changes in the urethra, alteration of the bladder wall structure, and detrusor function (9). Indeed, post-AUS incontinence may be seen owing to attenuated AUS cuff compression around the urethra caused by radiation-induced urethral atrophy. Incidence of atrophy may be further exacerbated by androgen deprivation which is frequently used in patients receiving RT (25). In contrast to this postulation, in our meta-analysis, we noted no difference in the incidence of urethral atrophy between RT and no-RT groups. One reason for this could be the small number of studies reporting data on urethral atrophy and the small sample size of studies included in the meta-analysis for this variable.

In our review, the risk of revision surgery was 24.1% in patients receiving RT and 17.4% amongst those not receiving RT with a statistically significant increased risk of revision surgery in patients with history of RT. Our results are consistent with the previous review (10), which also noted an increased risk of revision in patients with RT. However, contrasting results have been reported by other cohorts which were not included in this review due to a lack of detailed outcome data. The study of Radomski et al. (33) analyzing a cohort of 1,632 patients noted no impact of prior RT on the risk of revision/removal of AUS [Hazard ratio (HR): 1.01 95% CI: 0.80, 1.27]. We noted 2.5 times increased tendency of infection and a two-fold statistically significant increased risk of erosion in AUS patients with a history of RT as compared to those without RT. While the results for risk of infection were statistically non-significant due to wide CI, the upper end of the 95% CI was 6.29, indicating up to 6-times increased risk of infection in the RT group. Furthermore, on the exclusion of some studies, the results turned statistically significant indicating an increased risk of infection with RT. Our results assume significance as many of the included studies in the analysis have reported no difference in the risk of infection between the two groups and this may be due to the limited sample size of the studies. By combining data, the statistical power was significantly increased which could have contributed to the difference in the results. Our results are supported by the recent study of McKibben et al. (34) wherein history of pelvic RT was found to be an independent risk factor for erosions after AUS placement. It is suggested that progressive obliterating endarteritis and tissue atrophy are frequently noted with pelvic RT. The subsequent vascular compromise leads to urethral cuff erosion (35). However, it is important to note that several other factors can alter infection/erosion rates like patients' age, comorbidities like diabetes, prior urethral surgery, etc. These factors were not taken into consideration in the majority of the included studies. Our analysis also demonstrated increased risk of explanation in the RT group. However, these results should be interpreted with caution as data were pooled from just three studies and further corroboration is needed to strengthen this evidence.

Our review has several limitations. Firstly, most of the studies in the review were retrospective in nature. Such types of studies are prone to selection bias. Secondly, the overall quality of included studies was not high and bias due to several factors could have compromised the results. Thirdly, there were several methodological variations amongst the included studies like the etiology of UI, initial severity of UI, type of prostate surgery, the surgical technique of AUS placement, timing of RT, the dosage of RT, perioperative protocol, etc. all of which could have skewed outcomes. Baseline comparability of data by propensity score matching and reporting of multivariate-adjusted outcomes was not universally followed by the included studies. Furthermore, lack of data for such important confounding variables precluded a subgroup or meta-regression in our review. Fourthly, success and complications with surgical procedures also depend on expertise and surgical skills. This factor could not be considered in our review. Fifthly, the type of RT was not consistent across the included studies. Many of the studies did not specify whether RT was used as the primary treatment modality or used post-surgery as adjuvant or salvage therapy. In studies reporting data on mixed population, outcomes were not presented separately for primary RT and post-surgery RT which limited our ability to comprehensively assess the outcomes. While we attempted a subgroup analysis based on the type of RT, the number of studies included in each group were to few to derive strong conclusions. Lastly, the majority of the included studies were from the USA and other western countries. This limits the generalizability of the results to the worldwide population.

Despite these limitations, the strength of our review lies in including only more recent studies in the analysis to provide contemporary data to practicing clinicians. The majority of the older studies from the previous review (10) were excluded to judge the impact of current surgical techniques and patient protocols on AUS outcomes.

## Conclusion

Our meta-analysis of recent studies indicates that RT significantly reduces the odds of achieving complete continence after AUS placement. History of RT does not increase the risk of urethral atrophy or mechanical failure in patients with AUS. However, the risk of revision surgery, erosions and explanations is significantly increased in patients with RT with a non-significant but increased tendency of infections. The focus of future studies should be to assess if variables like patient comorbidities, prior surgery, surgical technique, dosage and timing of RT, cuff size, etc., alter the impact of RT on AUS outcomes.

## Data Availability Statement

The original contributions presented in the study are included in the article/Supplementary Materials, further inquiries can be directed to the corresponding author/s.

## Author Contributions

LZ and YX designed the project, involved in data collection and data analysis, and prepare the manuscript. YX edit the manuscript. Both authors read and approved the final manuscript.

## Conflict of Interest

The authors declare that the research was conducted in the absence of any commercial or financial relationships that could be construed as a potential conflict of interest.

## Publisher's Note

All claims expressed in this article are solely those of the authors and do not necessarily represent those of their affiliated organizations, or those of the publisher, the editors and the reviewers. Any product that may be evaluated in this article, or claim that may be made by its manufacturer, is not guaranteed or endorsed by the publisher.
