# Supplementary material for: Impact of Radiation Therapy on Outcomes of Artificial Urinary Sphincter: A Systematic Review and Meta-Analysis
Source: Front Surg. 2022 Feb 14;9:825239. doi: 10.3389/fsurg.2022.825239 (PMC8882597; doi:10.3389/fsurg.2022.825239)
Supplement: Supplementary file 1 [file Table_1.DOCX]

Supplementary Table 1: Search strategy

| **Search number** | **Query** | **Search Details** |
| --- | --- | --- |
| **1** | (Artificial urinary sphincter) AND (radiotherapy) | ("urinary sphincter, artificial"[MeSH Terms] OR ("urinary"[All Fields] AND "sphincter"[All Fields] AND "artificial"[All Fields]) OR "artificial urinary sphincter"[All Fields] OR ("artificial"[All Fields] AND "urinary"[All Fields] AND "sphincter"[All Fields])) AND ("radiotherapy"[MeSH Terms] OR "radiotherapy"[All Fields] OR "radiotherapies"[All Fields] OR "radiotherapy"[MeSH Subheading] OR "radiotherapy s"[All Fields]) |
| **2** | (Artificial urinary sphincter) AND (radiation) | ("urinary sphincter, artificial"[MeSH Terms] OR ("urinary"[All Fields] AND "sphincter"[All Fields] AND "artificial"[All Fields]) OR "artificial urinary sphincter"[All Fields] OR ("artificial"[All Fields] AND "urinary"[All Fields] AND "sphincter"[All Fields])) AND ("radiate"[All Fields] OR "radiated"[All Fields] OR "radiates"[All Fields] OR "radiating"[All Fields] OR "radiation"[MeSH Terms] OR "radiation"[All Fields] OR "electromagnetic radiation"[MeSH Terms] OR ("electromagnetic"[All Fields] AND "radiation"[All Fields]) OR "electromagnetic radiation"[All Fields] OR "radiations"[All Fields] OR "radiation s"[All Fields] OR "radiator"[All Fields] OR "radiators"[All Fields]) |
| **3** | (Artificial urinary sphincter) AND (EBRT) | ("urinary sphincter, artificial"[MeSH Terms] OR ("urinary"[All Fields] AND "sphincter"[All Fields] AND "artificial"[All Fields]) OR "artificial urinary sphincter"[All Fields] OR ("artificial"[All Fields] AND "urinary"[All Fields] AND "sphincter"[All Fields])) AND "EBRT"[All Fields] |
| **4** | (AMS800) AND (radiotherapy) | "AMS800"[All Fields] AND ("radiotherapy"[MeSH Terms] OR "radiotherapy"[All Fields] OR "radiotherapies"[All Fields] OR "radiotherapy"[MeSH Subheading] OR "radiotherapy s"[All Fields]) |
| **5** | (AMS800) AND (radiation) | "AMS800"[All Fields] AND ("radiate"[All Fields] OR "radiated"[All Fields] OR "radiates"[All Fields] OR "radiating"[All Fields] OR "radiation"[MeSH Terms] OR "radiation"[All Fields] OR "electromagnetic radiation"[MeSH Terms] OR ("electromagnetic"[All Fields] AND "radiation"[All Fields]) OR "electromagnetic radiation"[All Fields] OR "radiations"[All Fields] OR "radiation s"[All Fields] OR "radiator"[All Fields] OR "radiators"[All Fields]) |
