# Supplementary figures and images for: Impact of Radiation Therapy on Outcomes of Artificial Urinary Sphincter: A Systematic Review and Meta-Analysis
Source: Front Surg. 2022 Feb 14;9:825239. doi: 10.3389/fsurg.2022.825239 (PMC8882597; doi:10.3389/fsurg.2022.825239)

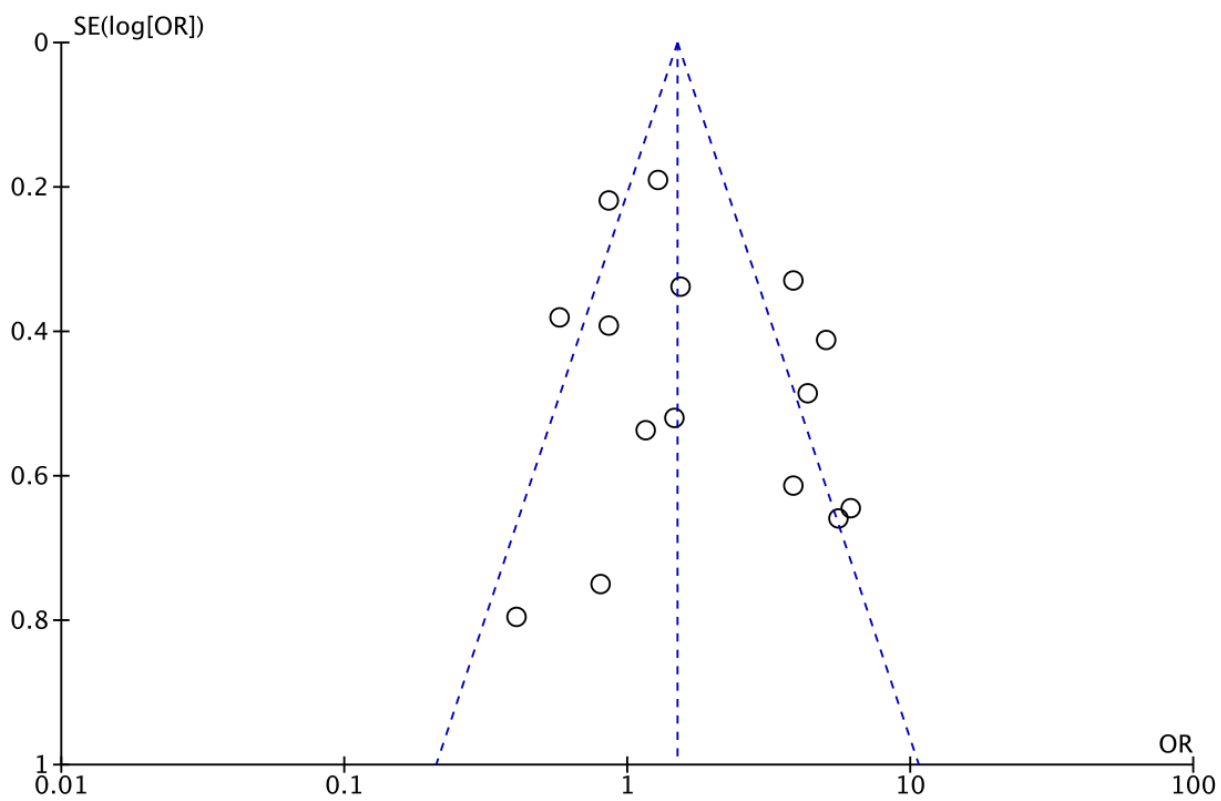

Supplement: Supplementary file 2 [file Data_Sheet_1.PDF]

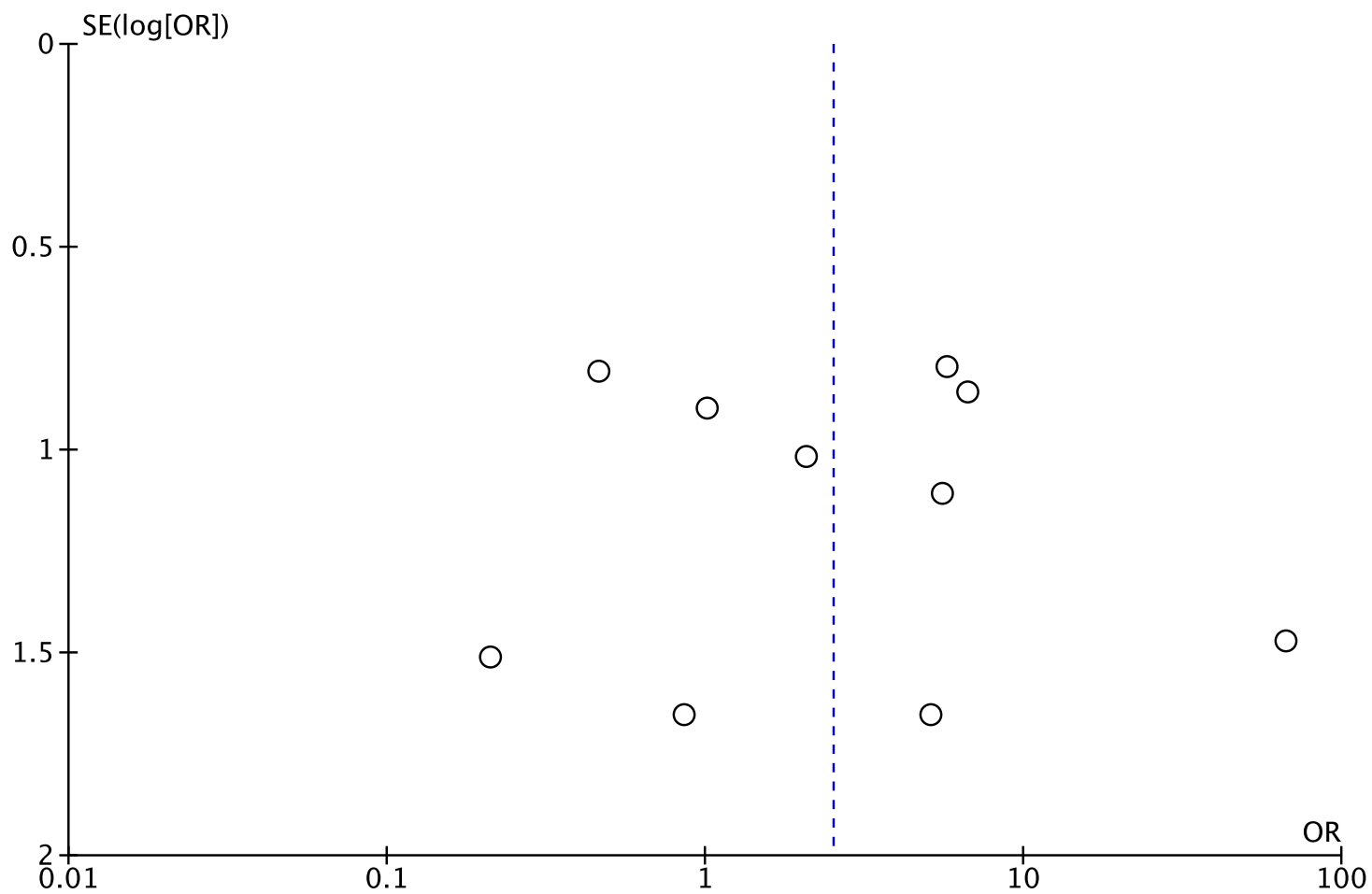

Supplement: Supplementary file 3 [file Data_Sheet_2.PDF]

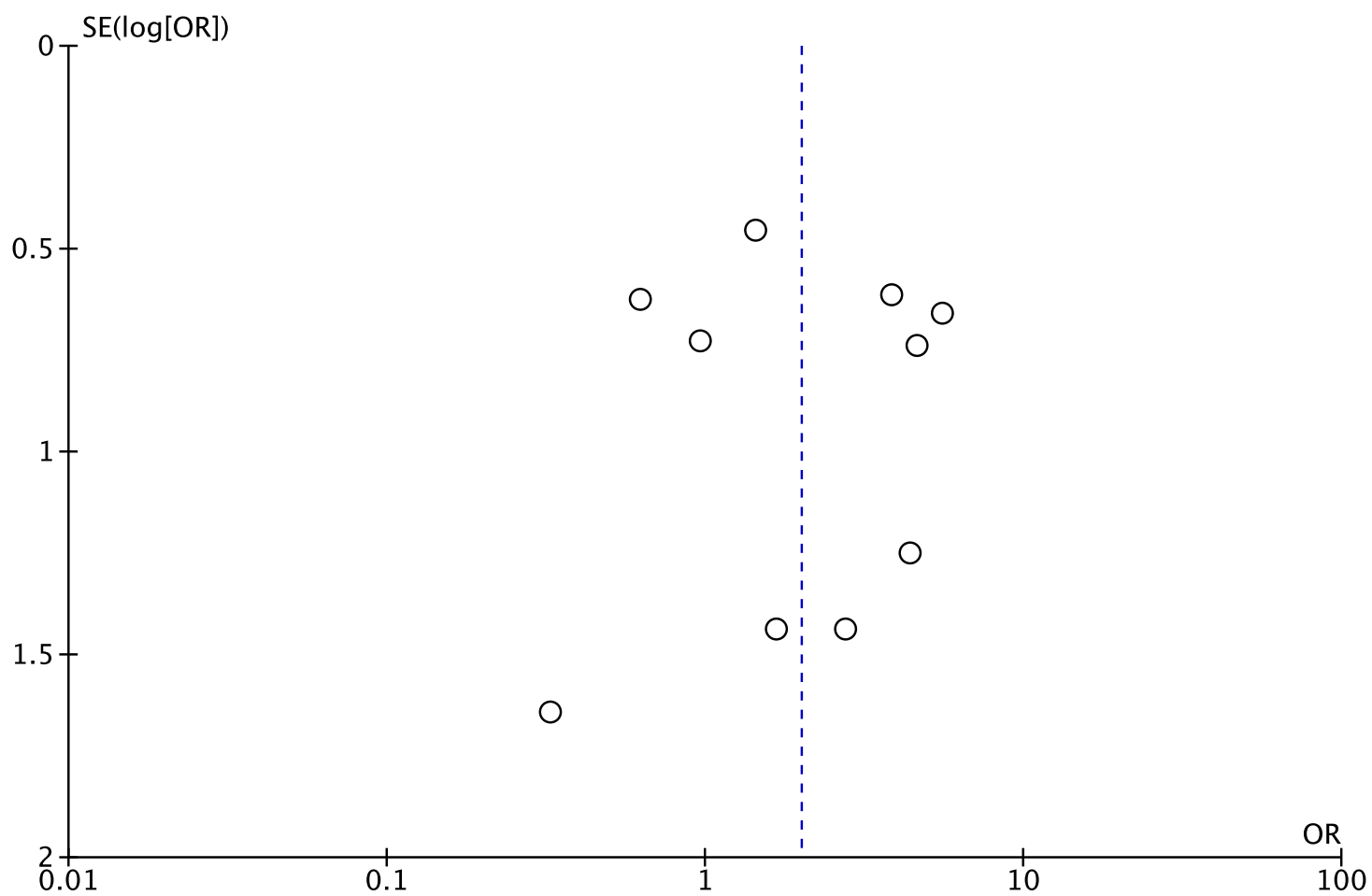

Supplement: Supplementary file 4 [file Data_Sheet_3.PDF]
